# Three cases of *mcr*-1-positive colistin-resistant *Escherichia coli* bloodstream infections in Italy, August 2016 to January 2017

**DOI:** 10.2807/1560-7917.ES.2017.22.16.30517

**Published:** 2017-04-20

**Authors:** Marta Corbella, Bianca Mariani, Carolina Ferrari, Francesco Comandatore, Erika Scaltriti, Piero Marone, Patrizia Cambieri

**Affiliations:** 1 SC Microbiologia e Virologia, Fondazione IRCCS Policlinico San Matteo, Pavia, Italy; 2 These authors contributed equally to this work; 3 Dipartimento di Biologia e Biotecnologie “L. Spallanzani”- Università degli Studi di Pavia, Pavia, Italy; 4 Centro di Ricerca pediatrico Romeo ed Enrica Invernizzi. Dipartimento di Scienze Biomediche e Cliniche L. Sacco, Università degli Studi di Milano, Milano, Italy; 5 Unità di Analisi del Rischio, Istituto Zooprofilattico Sperimentale della Lombardia ed Emilia Romagna, Parma, Italy

**Keywords:** mcr-1 E.coli, colistin resistance, blood stream infections, surveillance, public health, sepsis

## Abstract

We describe three cases of bloodstream infection caused by colistin-resistant *Escherichia coli* in patients in a tertiary hospital in Italy, between August 2016 and January 2017. Whole genome sequencing detected the *mcr-1* gene in three isolated strains belonging to different sequence types (STs). This occurrence of three cases with *mcr-1*-positive *E. coli* belonging to different STs in six months suggests a widespread problem in settings where high multidrug resistance is endemic such as in Italy.

A new plasmid-mediated transferable resistance determinant, the *mcr-1* gene, encoding a phosphoetha-nolamine transferase, has been described in November 2015 by Liu et al. for the first time [1,2]. The plasmid carrying *mcr-1* is mobilised to an *Escherichia coli* recipient by conjugation [1,2]. Since that description, the *mcr-1* gene has been detected in isolates recovered from animals, in the food chain and in humans in many countries in Europe and in many other areas worldwide [3-8].

Here we describe three cases of human bloodstream infection in Italy caused by *E. coli* harbouring the *mcr-1* gene. All three patients were hospitalised in a 1,000-bed hospital, in Pavia, in the period between August 2016 and January 2017.

## Case description

### Case 1

In July 2016, a woman in her 70s with a pancreatic ductal carcinoma diagnosed 5 years earlier, was admitted in a respiratory disease unit for pleural effusion. It was known that she had bone and liver metastases, was splenectomised, and had received 18 chemotherapy cycles in the previous 5 years. Four days after admission she experienced fever (38.8° C), vomiting and abdominal pain associated with increased inflammatory markers: procalcitonin 60 ng/mL (norm: 0.00–0.50 ng/mL), C-reactive protein (CRP) 23.29 mg/dL (norm: 0.00–0.50 mg/dL) and highly elevated white blood cells (WBC) 38.92 x 10^3^ uL (norm: 4–10 x 10^3^ uL). *E. coli* was isolated from urine and blood cultures. The two isolates showed the same antimicrobial susceptibility profiles. Both showed resistance to colistin. The susceptibly profile of the isolate obtained from blood is shown in the Table.

**Table:** Antibiotic susceptibility according to European Committee on Antimicrobial Susceptibility Testing (EUCAST) clinical breakpoints [9] of clinical *Eschericha coli* isolates obtained from human cases with bloodstream infections, Pavia, Italy, August 2016–January 2017 (n = 3 cases)

			MIC mg/L (S/I/R)												
**Case**	**Isolation date**	**Sequence type**	** CST**	** AMK**	** AMP**	** CAZ**	** CTX**	** FEP**	** CIP**	** FOS**	** MEM**	** GEN**	** TZP**	** SXT**	** TGC**
Case 1	7 Aug 2016	131	4 (R)	< 4 (S)	≤ 2 (S)	≤ 0.5 (S)	≤ 1 (S)	≤ 1 (S)	≤ 0.25 (S)	≤ 16 (S)	≤ 0.125 (S)	≤ 1 (S)	≤ 4/4 (S)	≤ 1/19 (S)	≤ 0.5 (S)
Case 2	12 Aug 2016	3941	4 (R)	< 4 (S)	> 8 (R)	≤ 0.5 (S)	≤ 1 (S)	≤ 1 (S)	> 1 (R)	≤ 16 (S)	≤ 0.125 (S)	> 4 (R)	≤ 4/4 (S)	> 4/76 (R)	≤ 0.5 (S)
Case 3	22 Jan 2017	1851	4 (R)	< 4 (S)	> 8 (R)	≤ 0.5 (S)	≤ 1 (S)	≤ 1 (S)	≤ 0.25 (S)	≤ 16 (S)	≤ 0.125 (S)	≤ 1 (S)	≤ 4/4 (S)	> 4/76 (R)	≤ 0.5 (S)

AMK: amikacin; AMP: ampicillin; CAZ: ceftazidime; CIP: ciprofloxacin; CST: colistin; CTX: cefotaxime; FEP: cefepime; FOS: fosfomycin; GEN: gentamicin; I: intermediate; MEM: meropenem; MIC: minimum inhibitory concentration; R: resistant; S: susceptible; SXT: trimethoprim-sulfamethoxazole; TGC: tigecycline; TZP: piperacillin–tazobactam.

The patient was empirically treated with meropenem intravenously and her clinical condition rapidly improved. She was discharged after 20 days of hospitalisation. She had never received previous treatment with colistin and did not report any previous close contacts with farm animals. She had not travelled abroad since 2008.

### Case 2

In August 2016, a woman in her mid-60 with a diagnosis of non-Hodgkin’s Lymphoma was admitted at the haematological unit of our hospital for distention of the ureter, renal pelvis and calices due to blockage of urine flow by bulky lymph nodes. She had been treated with two cycles of chemotherapy in the previous 6 weeks with poor response. She underwent nephrostomy after admission and one day later she developed fever (38.0° C) and chills. She had a severe pancytopenia with less than 0.800 x 10^3^/uL (norm: 4–10 x 10^3^ uL) WBC and increase of CRP 3.50 mg/dL (0.00–0.50 mg/dL).

The patient received intravenous empirical treatment with piperacillin/tazobactam and vancomycin. *E. coli* was isolated from urine and blood cultures. As with Case 1, the two isolates obtained showed the same antimicrobial susceptibility profiles, both indicating resistance to colistin. The susceptibility profile of the isolate obtained from blood is shown in the Table.

The CRP decreased to normal range within 3 days and the fever disappeared within 24 hours. The patient died 5 days later due to a massive cerebral haemorrhage. She had never received previous treatment with colistin and did not report any previous close contact with farm animals. In the previous 12 years she had not travelled abroad.

### Case 3

In January 2017, a woman in her early 80s with fever (> 38.5° C), diarrhoea and abdominal pain was admitted at the infectious diseases unit of the same tertiary hospital. In 2012, she underwent mastectomy and chemotherapy for breast cancer. Blood cultures were drawn upon admission and she received intravenous empirical treatment with piperacillin/tazobactam. *E. coli* was isolated from blood cultures. The Table shows the antimicrobial profile; the strain was colistin-resistant but susceptible to other commonly used antimicrobials (Table). She had 10.03 x 10^3^/uL WBC (norm: 4–10 x 0^3^ uL) and high inflammatory markers: procalcitonin 50.50 ng/mL (norm: 0.00–0.5 ng/mL), CRP 28.41 mg/dL (norm: 0.00–0.5 mg/dL).

Her clinical condition rapidly improved and she was discharged from hospital after 8 days. She had never been treated with colistin, did not report any previous close contact with farm animals and she had never travelled abroad.

## Microbiological findings

Blood samples for cultures were collected in BD BACTEC culture aerobic/anaerobic vials and were incubated into BACTEC FX automated blood culture system (Becton Dickinson and Company, Franklin Lakes, New Jersey, United States), according to the manufacturer’s instructions.

Positive blood cultures were subjected to Gram-staining and subcultured into aerobic sheep blood agar plates, chocolate agar plates, selective plates and into Schaedler agar and 5% sheep blood plates (bioMérieux SA, Marcy-l’Etoile, France) anaerobically and incubated at 37°C overnight: the organisms were identified by Matrix-Assisted Laser Desorption Ionization time-of-flight (MALDI-TOF) (Bruker Daltonics GmbH, Bremen, Germany).

The isolates were tested for antimicrobial susceptibility using Phoenix 100 (BD) automated system N-MIC panel. Isolates flagged positive for colistin resistance by the system were further tested according to European Committee on Antimicrobial Susceptibility Testing (EUCAST) clinical breakpoints (version 6.0) [9]. As recommended by EUCAST [10] and the European Centre for Disease Prevention and Control (ECDC) [3], minimal inhibitory concentration (MIC) of colistin was determined by broth microdiluition (UMIC colistine ARNIKA SRL Diagnostic Line, Milano, Italy). The MIC of colistin was 4 mg/L for all the isolates (≤2 susceptible, >2 resistant).

Only blood isolates were further tested: unfortunately, the urine ones, which had a similar resistance profile to the isolates from blood, were not available for investigation.

Whole genome DNA was extracted from each isolate using a QIAamp DNA minikit (Qiagen) following the manufacturer’s instructions, and sequenced using an Illumina Miseq platform with a 2 by 250 paired-end run after Nextera XT paired-end library preparation.

Genome assembly was performed using SPAdes-3.10.1 software. The genomic sequences were submitted to the European Nucleotide Archive (ENA) (accession numbers pending). Multilocus sequence typing (MLST) profiles were obtained in silico by analysing appropriate gene variants for each genome, using an in-house Perl script, based on the Achtman MLST scheme [11]. Analysis of the 7-gene MLST showed that the three isolates belong to three different sequence types: ST131, ST3941 and ST1851 respectively.

All three *E. coli* isolates obtained from blood were colistin-resistant. We thus searched the genomes for the presence of *mcr-1* and *mcr-2* genes as both these genes can confer plasmid-mediated resistance to colistin. A Basic Local Alignment Search Tool (BLAST) searches of *mcr-1* (plasmid accession number KP347127) and mcr-2 (LT598652) against the three assemblies were performed. For each genome we obtained a best hit for *mcr-1* gene with a very low number of nucleotide (nt) differences (2, 0 and 1 nt differences for the ST131, ST3941 and ST1851 strains, respectively) and no best hit for mcr-2. Considering that *mcr-1* and *mcr-2* gene sequences used in the analysis are very different at nt level (375 nt differences), the results show that all three genomes harboured *mcr-1* and none harboured *mcr-2*.

## Discussion

The occurrence of colistin resistance based on the plasmid-encoded *mcr-1* gene in *Enterobacteriaceae* has been described in different European countries since it was first reported in November 2015 [3-8].

To our knowledge, our data show the first three bloodstream infections mediated by *mcr-1*-encoding *E. coli* in Italy [12,13]. A limitation of our report is that we did not perform a systematic investigation of all bloodstream infections over a certain period in our hospital and we are thus lacking denominator data that would provide better insight into the frequency of the problem. A study is planned to retrospectively analyse all *E. coli* strains obtained in the past year in our hospital to complete our data.

All three patients in our case series had underlying oncological diseases with different degrees of severity which would put them at higher risk of sepsis, however not at higher risk of exposure to *mcr-1*–positive pathogens. None of the patients was previously exposed to colistin. Except for being hospitalised at the same institution at different points in time over a 6-month period, no other epidemiological link could be determined between them. Moreover, the three *mcr-1*-positive isolates belonged to three different STs, indicating the presence of different colistin-resistant strains.

The first of the three strains belonged to ST131. This ST has been described by Overdevest et al. as an *E. coli* clone associated with extended-spectrum beta-lactamase (ESBL) production that can colonise patients for prolonged periods, with an estimated half-life of 13 months [14] and by Wang et al. as a prevalent ST in China probably associated with a high risk of dissemination among *E. coli* [6]. In our patient, the ST131 *E. coli* did not appear to be more pathogenic.

The ST1851 is a new ST, and the draft genome assembly of the respective *E. coli*-1851 strain has been deposited at ENA (accession numbers pending).

All three isolates described here showed a favourable susceptibility profile to other classes of antimicrobials and the three bloodstreams infections were rapidly resolved with the chosen empirical therapy. In a recently published article, Poirel et al. [15] describe how not all plasmids with an *mcr-1* gene carry other genes encoding resistance to clinically relevant antibiotics, such as β-lactams, aminoglycosides, quinolones, fosfomycin, sulfonamides, and tetracyclines. In 2016, Bernasconi et al. [16] and Prim et al. [17] also reported occurrence of the *mcr-1* gene in pathogens without presence of further genes conferring resistance for example to extended-spectrum cephalosphorins or carbapenemase. This fact may account for the positive outcome of the infections described here but on the other side further highlights the importance of horizontal dissemination of *mcr-1* gene-related colistin resistance in non-multidrug-resistant (MDR) *E. coli* isolates of human origin.

The identification of three patients with bloodstream infections caused by different strains of *mcr-1*–positive *E. coli* detected within 6 months in a single hospital suggests an important and widespread problem [3]. Furthermore, our findings suggest that the dissemination of *mcr-1*-positive *E. coli* in Italy could be underestimated because isolates may be susceptible to other tested antibiotics and screening is often focused on carbapenem-resistant strains only. Moreover, in Italy, where the MDR bacteria are endemic, the acquisition of *mcr-1* plasmid-mediated genes by other MDR *Enterobacteriaceae* could lead to a severe public health concern because it seriously limits treatment options as already reported in 2016 by Di Pilato et al. [18]. Our data further highlight the need of strict surveillance of colistin resistance even in multi-susceptible isolates.

## References

[1] Liu YY Wang Y Walsh TR Yi LX Zhang R Spencer J Emergence of plasmid-mediated colistin resistance mechanism MCR-1 in animals and human beings in China: a microbiological and molecular biological study. Lancet Infect Dis. 2016;16(2):161-8. 10.1016/S1473-3099(15)00424-7 26603172

[2] Li J Nation RL Turnidge JD Milne RW Coulthard K Rayner CR Colistin: the re-emerging antibiotic for multidrug-resistant Gram-negative bacterial infections. Lancet Infect Dis. 2006;6(9):589-601. 10.1016/S1473-3099(06)70580-1 16931410

[3] European Centre for Disease Prevention and Control (ECDC). Plasmid-mediated colistin resistance in Enterobacteriaceae. Stockolm: ECDC; 2016. Available from: http://ecdc.europa.eu/en/publications/Publications/enterobacteriaceae-risk-assessment-diseases-caused-by-antimicrobial-resistant-microorganisms-europe-june-2016.pdf

[4] Walkty A Karlowsky JA Adam HJ Lagacé-Wiens P Baxter M Mulvey MR Frequency of MCR-1-mediated colistin resistance among Escherichia coli clinical isolates obtained from patients in Canadian hospitals (CANWARD 2008-2015). CMAJ Open. 2016;4(4):E641-5. 10.9778/cmajo.20160080 28018876 PMC5173483

[5] Quan J, Li X, Chen Y, Jiang Y, Zhou Z, Zhang H, et al. Prevalence of mcr-1 in Escherichia coli and Klebsiella pneumoniae recovered from bloodstream infections in China: a multicentre longitudinal study. Lancet Infect Dis. 2017;17(4):400-10. 28139430 10.1016/S1473-3099(16)30528-X

[6] Wang Y Tian GB Zhang R Shen Y Tyrrell JM Huang X Prevalence, risk factors, outcomes, and molecular epidemiology of mcr-1-positive Enterobacteriaceae in patients and healthy adults from China: an epidemiological and clinical study. Lancet Infect Dis. 2017;17(4):390-9. 10.1016/S1473-3099(16)30527-8 28139431

[7] Nordmann P Lienhard R Kieffer N Clerc O Poirel L Plasmid-Mediated Colistin-Resistant Escherichia coli in Bacteremia in Switzerland. Clin Infect Dis. 2016;62(10):1322-3. 10.1093/cid/ciw124 26936673

[8] Skov RL Monnet DL Plasmid-mediated colistin resistance (mcr-1 gene): three months later, the story unfolds. Euro Surveill. 2016;21(9):30155. 10.2807/1560-7917.ES.2016.21.9.30155 26967914

[9] European Committee on Antimicrobial Susceptibility Testing (EUCAST). Breakpoint tables for interpretation of MICs and zone diameters. Version 6.0, valid from 2016-01-01. Available from: http://www.eucast.org/fileadmin/src/media/PDFs/EUCAST_files/Breakpoint_tables/v_6.0_Breakpoint_table.pdf

[10] European Committee on Antimicrobial Susceptibility Testing (EUCAST). Recommendations for MIC determination of colistin (polymyxin E). As recommended by the joint CLSI-EUCAST Polymyxin Breakpoints Working Group. Växjö: EUCAST; 22 Mar 2016. Available from: http://www.eucast.org/fileadmin/src/media/PDFs/EUCAST_files/General_documents/Recommendations_for_MIC_determination_of_colistin_March_2016.pdf

[11] Wirth T Falush D Lan R Colles F Mensa P Wieler LH Sex and virulence in Escherichia coli: an evolutionary perspective. Mol Microbiol. 2006;60(5):1136-51. 10.1111/j.1365-2958.2006.05172.x 16689791 PMC1557465

[12] Cannatelli A Giani T Antonelli A Principe L Luzzaro F Rossolini GM First Detection of the mcr-1 Colistin Resistance Gene in Escherichia coli in Italy. Antimicrob Agents Chemother. 2016;60(5):3257-8. 10.1128/AAC.00246-16 26976865 PMC4862502

[13] Giufrè M Monaco M Accogli M Pantosti A Cerquetti M PAMURSA Study Group Emergence of the colistin resistance mcr-1 determinant in commensal Escherichia coli from residents of long-term-care facilities in Italy. J Antimicrob Chemother. 2016;71(8):2329-31. 10.1093/jac/dkw195 27261262

[14] Overdevest I Haverkate M Veenemans J Hendriks Y Verhulst C Mulders A Prolonged colonisation with Escherichia coli O25:ST131 versus other extended-spectrum beta-lactamase-producing E. coli in a long-term care facility with high endemic level of rectal colonisation, the Netherlands, 2013 to 2014. Euro Surveill. 2016;21(42):30376. 10.2807/1560-7917.ES.2016.21.42.30376 27784530 PMC5291152

[15] Poirel L Jayol A Nordmann P Polymyxins: Antibacterial Activity, Susceptibility Testing, and Resistance Mechanisms Encoded by Plasmids or Chromosomes. Clin Microbiol Rev. 2017;30(2):557-96. 10.1128/CMR.00064-16 28275006 PMC5355641

[16] Bernasconi OJ Kuenzli E Pires J Tinguely R Carattoli A Hatz C Travelers can import colistin resistant Enterobacteriaceae, including those possessing the plasmid-mediated mcr-1 gene. Antimicrob Agents Chemother. 2016;60(8):5080-4. 10.1128/AAC.00731-16 27297483 PMC4958239

[17] Prim N Rivera A Rodríguez-Navarro J Español M Turbau M Coll P Detection of mcr-1 colistin resistance gene in polyclonal Escherichia coli isolates in Barcelona, Spain, 2012 to 2015. Euro Surveill. 2016;21(13):30183. 10.2807/1560-7917.ES.2016.21.13.30183 27055477

[18] Di Pilato V Arena F Tascini C Cannatelli A Henrici De Angelis L Fortunato S mcr-1.2, a New mcr Variant Carried on a Transferable Plasmid from a Colistin-Resistant KPC Carbapenemase-Producing Klebsiella pneumoniae Strain of Sequence Type 512. Antimicrob Agents Chemother. 2016;60(9):5612-5. 10.1128/AAC.01075-16 27401575 PMC4997870

